# The therapeutic effect of omega-3 polyunsaturated fatty acids on symptom severity of psychosis: A systematic review and meta-analysis

**DOI:** 10.1192/j.eurpsy.2024.1804

**Published:** 2024-12-19

**Authors:** Alison T. Rossier, Brian Hallahan

**Affiliations:** 1 Department of Pharmacology and Therapeutics, College of Medicine, Nursing and Health Sciences, University of Galway, Galway, Ireland; 2 Department of Psychiatry, College of Medicine, Nursing and Health Sciences, University of Galway, Galway, Ireland

**Keywords:** early psychosis intervention, meta-analysis, omega-3 polyunsaturated fatty acids, psychotic disorders, schizophrenia, systematic review

## Abstract

**Background:**

While omega-3 polyunsaturated fatty acids (PUFAs) have shown promise as an adjunctive treatment for schizophrenia and other psychotic disorders, the overall consensus about their efficacy across studies is still lacking and findings to date are inconclusive. No clinical trials or systematic reviews have yet examined if omega-3 PUFAs are associated with differential levels of efficacy at various stages of psychosis.

**Method:**

A systematic bibliographic search of randomized double-blind placebo-controlled trials (RCTs) examining the effect of omega-3 PUFAs as a monotherapy or adjunctive therapy versus a control group in adults and children at ultra-high risk (UHR) for psychosis, experiencing a first-episode psychosis (FEP), or diagnosed with an established psychotic disorder was conducted. Participants’ clinical symptoms were evaluated using total and subscale scores on validated psychometric scales.

**Results:**

No beneficial effect of omega-3 PUFAs treatment was found in comparison with that of placebo (*G* = −0.26, 95% CI −0.55 to 0.03, *p* = 0.08). Treatment of omega-3 PUFAs did not prove any significant improvement in psychopathology in UHR (*G* = −0.09, 95% CI −0.45 to 0.27, *p* = 0.63), FEP (*G* = −1.20, 95% CI −5.63 to 3.22, *p* = 0.59), or schizophrenia patients (*G* = −0.17, 95% CI −0.38 to −0.03, *p* = 0.10).

**Conclusion:**

These findings confirm previous evidence that disputes the original reported findings of the beneficial effect of omega-3 PUFAs in schizophrenia. Furthermore, accumulative evidence of the use of omega-3 as a preventive treatment option in UHR is not supported, suggesting that the need for future studies in this line of research should not be promoted.

## Introduction

Psychotic disorders such as schizophrenia are frequently severe and disabling and associated with poor functional outcomes [1, 2]. Given the sometimes-modest treatment response associated with antipsychotic agents, novel approaches including omega-3 polyunsaturated fatty acids (PUFAs) have been postulated as potential novel therapeutic options. Initial interest in omega-3 PUFAs derived from documented abnormalities of the phospholipid membrane in schizophrenia and other psychotic disorders, with Horrobin and colleagues, suggesting that dysfunctional fatty acid metabolism could be of aetiological significance [3]. Considering the importance of phospholipids for neuronal functioning, alterations in phospholipid membrane may cause secondary abnormalities in various neurotransmitters, ion channels, and cell signalling systems due to changes in protein structures and cell signalling mechanisms [3, 4]. Correction of abnormal membrane structure by targeting modulatory activities involved in phospholipid metabolism using omega-3 PUFAs has therefore been suggested [5]. Due to their role in phospholipid synthesis, enzyme regulation, and membrane modulation, omega-3 PUFAs may potentially prevent biochemical changes observed in psychotic disorders. For example, reduced levels of omega-3 PUFAs, in particular, docosahexaenoic acid (DHA) in peripheral blood plasma and erythrocyte membranes of schizophrenia patients at different development stages [6, 7] (individuals at ultra-high risk (UHR) for psychosis [8], unmedicated first-episode psychosis (FEP) [9], and chronic patients [10]) have been reported. Moreover, the breakdown of phospholipids and reduction of DHA in the brain orbitofrontal cortex has been demonstrated in psychotic patients [11], suggesting a potential association between a deficit of omega-3 PUFAs, including DHA deficit and the pathogenesis of psychotic disorders. Clinical trials examining the efficacy of omega-3 PUFAs in psychotic disorders have however provided variable results. Potential reasons for this variability include the utilization of different doses or formulations of omega-3 PUFAs (including either eicosapentaenoic acid (EPA) or DHA predominant formulations) and the conduct of trials in different subgroups of patients with psychosis (UHR, FEP, and chronic schizophrenia). Despite some initial studies demonstrating a reduction in transition to psychosis in individuals at UHR for psychosis [12, 13], not all studies have subsequently replicated these findings [14]. For individuals experiencing a FEP, omega-3 PUFA supplementation has been associated with a reduction in psychotic symptoms [15-18], although again these findings have not been universally replicated [19]. Among chronic schizophrenia patients, variable results have also been noted. For example, an amelioration of symptoms has been demonstrated in patients diagnosed with treatment-resistant schizophrenia on clozapine after omega-3 PUFA supplementation [20]; however, a number of other studies have demonstrated no significant clinical improvement in chronic patients compared to healthy controls after omega-3 treatment [21–23]. A number of previous systematic reviews and meta-analyses have been conducted to ascertain the efficacy of omega-3 PUFA supplementation for psychotic symptoms [24–27]. Most consist of a modest number of studies and participants, with variable findings. No improvement with omega-3 PUFA supplementation has been noted with EPA and EPA/DHA supplementation in schizophrenia [25–27] with the exception of the largest previous meta-analysis consisting of 13 studies that noted an improvement in overall symptoms (standard mean difference (SMD) = −0.27, 95% CI −0.41, −0.14, *p* < 0.001) [24]. Beneficial effects were also noted for individuals at UHR for psychosis or experiencing a FEP; however, only two studies were analyzed for both these groups [24] with other reviews noting no benefit of omega-3 PUFAs in individuals at UHR for psychosis albeit low numbers of participants were included in analyses [28, 29].

Consequently, there is a lack of clarity in relation to the potential therapeutic benefit of omega-3 PUFAs across the spectrum of psychotic disorders. Thus, this systematic review and meta-analysis including all previously published RCTs explores if omega-3 PUFAs exhibit (1) a therapeutic benefit in psychotic disorders, (2) greater efficacy at different stages of psychosis (UHR v. FEP v. chronic schizophrenia), and (3) a differential impact on positive compared to negative symptoms.

## Method

### Data sources

The systematic review was conducted in line with the Preferred Reporting Items for Systematic Reviews and Meta-Analyses (PRISMA) guidelines [30]. The PRISMA checklist is presented in Supplementary Table S1. The protocol for the systematic review was registered on PROSPERO, the National Institute of Health Research Database (Registration Number: CRD42023438350).

A manual systematic electronic search of studies utilizing omega-3 PUFAs in psychotic disorders was conducted through the following databases: Medline, Embase, and Cochrane Central Register of Controlled Trials (CENTRAL). The search included all relevant articles published until November 2024, without language restrictions. The following subject heading keywords were used to find all relevant articles: psychosis OR psychotic disorder(s) OR schizophrenia OR schizophrenia spectrum OR non-affective disorder(s) OR FEP OR UHR for psychosis OR at-risk mental state (ARMS) AND omega-3 (n-3/ω-3) fatty acids OR essential fatty acids (EFAs) OR PUFAs OR EPA OR DHA OR fish oil OR nutritional supplement. A manual search was further performed for the above references from the papers identified, relevant reviews, Trials Central (http://www.trialscentral.org), the ISRCTN (http://controlled-trials.com), and Clinical Trials (http://clinicaltrials.gov) registries.

### Study selection

Double-blind placebo-controlled studies examining the therapeutic effect of omega-3 PUFAs on psychotic symptoms either as a monotherapy or adjunctive therapy in adults and children with psychosis (UHR for psychosis, FEP, or established schizophrenia) either as a primary or secondary outcome were included. Unblinded, single-blind, open-label, and pilot studies were excluded. Studies examining the effect of omega-3 PUFAs on various neurochemical, biochemical, and biological compounds were excluded. Substance and/or medication-induced psychotic disorders and disorders where psychotic symptoms were a consequence of a mood disorder (i.e. affective psychotic disorders: bipolar disorder, major depressive disorder) were additionally excluded. A diagnosis of a psychotic illness required the utilization of operational criteria including the Diagnostic and Statistical Manual of Mental Disorders (DSM) – IV [31] or the International Classification of Disease (ICD) – 10 [32] for FEP and schizophrenia, or the Comprehensive Assessment of At-Risk Mental States (CAARMS) [33], or Structured Interview for Psychosis Risk Syndromes (SIPS) [34], for individuals with UHR for psychosis. For all studies, symptomatic assessment was either considered a primary or secondary outcome measure.

### Data extraction

Two reviewers (A.R., B.H.) independently assessed and extracted relevant data including participants’ age, diagnosis, and type of antipsychotic treatment, trial duration, baseline psychometric scores and/or difference in scores between baseline and follow-up review(s) along with type, dosage, and prescription schedule of intervention. Corresponding authors of eligible studies were contacted by email in the event of incomplete or partly unavailable results. The following data were extracted by reviewers from July 8, 2023, and manually entered into a Microsoft Excel worksheet, later adapted into a table to visually display the results of individual studies (Table 1). Our primary analysis selected the following hierarchy of psychometric instruments: the Positive and Negative Syndrome Scale (PANSS; *n* = 21), the Brief Psychiatric Rating Scale (BPRS; *n* = 3), and the CAARMS (*n* = 1).

**Table 1.** Characteristics of randomized placebo-controlled trials of omega-3 polyunsaturated fatty acids on symptom severity of psychosis in UHR population and schizophrenia patients.

Study reference	Population	Age	Study duration	Monotherapy/adjunctive antipsychotics	Treatment dosage	Inactive antioxidant (vitamin E)	N	Psychometric scale/subscale	Mean (SD) pre/post treatment	Mean (SD) change from baseline	Efficacy^a^		
											T	P	N
Fenton et al., 2001	Schizophrenia or schizoaffective outpatients added to stable dose of antipsychotics	18–65	16 weeks	Unspecified antipsychotics	EPA 3.0g Placebo (mineral oil)	N/A N/A	43 44	Total PANSS	74.0(16.0)/69.0(16.0) 76.0(18.0)/70.0(18.0)	−5.0(16.0) −6.0(18.0)	ns	ns	ns
Peet et al., 2001a^d^	Schizophrenia outpatients added to stable dose of antipsychotics	–	12 weeks	Unspecified antipsychotics	EPA 2.0g DHA 2.0g Placebo (corn oil)	N/A N/A N/A	15 16 14	Total PANSS Positive PANSS	69.9(12.9) /55.5(12.2) 73.4(17.9) /65.3(19.0) 76.2(20.6) /65.9(14.9) 18.9(5.4)/14.6(5.9) 17.8(5.4)/16.7(5.3) 18.7(5.7)/15.8(5.1)	−14.4(12.6) −8.1(18.5) −10.3(18.4) −4.3(5.7) −1.1(5.4) −2.9(5.4)	s	s	ns
Peet et al., 2001b^d^	New onset or relapse schizophrenia outpatients added to flexible dose of antipsychotics	–	12 weeks	Conventional antipsychotics	EPA 2.0g Placebo (corn oil)	N/A N/A	14 12	Total PANSS Positive PANSS	70.4(10.1)/44.6(8.7) 79.3(18.6)/57.1(15.5) 23.1(8.7)/12.5(2.8) 24.7(8.2)/17.7(8.6)	−25.8(9.5) −22.2(17.3) −10.6(7.7) −7.0(8.4)	s	s	–
Peet & Horrobin., 2002	Schizophrenia outpatients added to stable dose of clozapine	18–70	12 weeks	Clozapine	EPA 1.0g EPA 2.0g EPA 4.0g Placebo (paraffin oil)	N/A N/A N/A N/A	29 28 27 31	Total PANSS	75.0(11.5)/–(–) 83.0(18.5)/– (–) 79.0(13.5)/– (–) 78.0(20.3)/– (–)	– (–) – (–) – (–) – (–)	s	s	s
Emsley et al., 2002	Schizophrenia outpatients added to flexible dose of antipsychotics	18–55	12 weeks	Unspecified antipsychotics	EPA 3.0g Placebo (paraffin oil)	N/A N/A	20 20	Total PANSS	76.0(–)/63.4(–) 74.0(–)/71.0(–)	−12.6(14) −3.1(13.3)	s	–	–
Emsley et al., 2006	Schizophrenia or schizoaffective inpatients with tardive dyskinesia added to stable dose of antipsychotics	18–60	12 weeks	Unspecified antipsychotics	EPA 2.0g Placebo (paraffin oil)	N/A N/A	39 38	Total PANSS	59.2(13.0)/– (–) 57.5(11.8)/– (–)	– (–) – (–)	ns	–	–
Berger et al., 2007	First-episode schizophrenia inpatients/outpatients added to flexible dose of antipsychotics	15–29	12 weeks	Atypical antipsychotics	EPA 2.0g Placebo (oil)	0.2% N/A	35 34	Total PANSS	109.6(14.6)/– (–) 111.8(21.8)/– (–)	– (–) – (–)	s	–	–
Manteghiy et al., 2008	Schizophrenia inpatients added to stable dose of risperidone	18–55	6 weeks	Risperidone	EPA 1.08g + DHA 0.72g Placebo (identical capsule)	N/A N/A	42 43	Total PANSS Positive PANSS Negative PANSS	149.5(8.1)/115.4(7.3) 147.8(8.2)/116.7(7.9) 50.4(7.6)/39.4(7.3) 49.0(7.7)/39.0(6.5) 48.0(8.1)/37.2(6.8) 46.8(8.1)/38.6(9.0)	−34.1(7.7) −31.1(8.1) −11.0(7.5) −10.0(7.2) −10.8(7.5) −8.2(8.6)	ns	ns	ns
Amminger et al., 2010	UHR outpatients on monotherapy	13–25	12 weeks	Monotherapy	EPA 0.7g + DHA 0.48g Placebo (coconut oil)	7.6mg N/A	34 33	Total PANSS Positive PANSS Negative PANSS	59.9(13.1)/–(–) 57.2(13.9)/– (–) 15.0(3.4)/– (–) 14.2(3.1)/– (–) 14.1(5.3)/– (–) 13.6(6.5)/– (–)	−15.7(16.3) −4.4(16.1) −4.4(4.7) −1.5(4.6) −3.1(7.04) 0.4(7.0)	s	s	s
Faghihi et al., 2012	Schizophrenia, schizoaffective, or bipolar unspecified patients added to stable dose of olanzapine and lithium/valproate	18–60	6 weeks	Olanzapine + lithium/valproate	EPA 0.9g Placebo (identical capsule)	N/A N/A	20 21	Total PANSS	– (–)/– (–) – (–)/– (–)	– (–) – (–)	–	–	–
Bentsen et al., 2013	Schizophrenia, schizoaffective, or schizophreniform inpatients added to stable dose of antipsychotics	18–39	16 weeks	Unspecified antipsychotics	EPA 2.0g EPA 2.0g Placebo (paraffin oil) Placebo (paraffin oil)	4mg 544IU^c^ 4mg 544IU^c^	33 18 25 28	Total PANSS Positive PANSS Negative PANSS	78.0(11.9)/– (–) 94.5(22.2)/– (–) 82.5(14.1)/– (–) 78.5(22.2)/– (–) 21.0(–)/– (–) 23.5(–)/– (–) 20.0(–)/– (–) 18.0(–)/– (–) 19.5(–)/– (–) 26.0(–)/– (–) 19.5(–)/– (–) 21.0(–)/– (–)	−17.3(12.5) −25.8(11.5) −23.5(9.2) −18.5(12.9) −4.6(4.05) −6.6(3.9) −8.0(2.4) −7.2(3.9) −4.7(6.1) −7.08(6.6) −3.4(6.06) −4.4(6.5)	sdc	sdc	–
Emsley et al., 2014	In remission first-episode schizophrenia, schizoaffective or schizophreniform outpatients with antipsychotics discontinued within 6 months	18–48	2 years	Monotherapy	EPA 2.0g + DHA 1.0g^e^ Placebo (olive oil)	N/A N/A	21 12	Total PANSS Positive PANSS Negative PANSS	36.1(4.2)/–(-) 38.2(4.0)/– (–) – (–)/– (–) – (–)/– (–) – (–)/– (–) – (–)/– (–)	−4.9(11.4) −16.3(8.8) 1.2(4.8) 0.5(3.9) −3.1(4.8) −7.1(3.0)	ns	ns	s
Jamilian et al., 2014	Schizophrenia unspecified patients started concurrently with standard antipsychotics	15–55	8 weeks	Atypical antipsychotics	EPA 1.0g Placebo (identical capsule)	N/A N/A	30 30	Total PANSS Positive PANSS Negative PANSS	96.1(9.6)/49.1(5.3) 98.3(4.5)/52.4(3.3) 26.7(3.3)/14.0(2.8) 27.6(3.9)/14.7(2.5) 23.8(3.4)/12.1(2.6) 23.06(3.6)/11.3(2.8)	−47.0(6.4) −45.8(2.8) −12.6(2.09) −13.0(2.5) −11.7(2.7) −11.8(3.0)	s	ns	ns
Amminger et al., 2015^b^	UHR outpatients on monotherapy	13–25	7 years	Monotherapy	EPA 0.7g + DHA 0.48g Placebo (coconut oil)	7.6mg N/A	28 29	Total PANSS Positive PANSS Negative PANSS	59.9(14.8)/–(–) 57.2(15.1)/– (–) 15.0(3.7)/– (–) 14.2(3.8)/– (–) 14.0(4.8)/– (–) 13.6(4.8)/– (–)	−13.9(17.5) 0.2(17.8) −5.1(4.8) −0.8(4.8) −3.1(5.8) 0.4(5.9)	s	s	s
Pawełczyk et al., 2016	First-episode schizophrenia inpatients added to flexible dose of antipsychotics	16–35	26 weeks	Unspecified antipsychotics	EPA 1.32g + DHA 0.88g Placebo (olive oil)	0.2% N/A	36 35	Total PANSS Positive PANSS Negative PANSS	98.4(13.2)/– (–) 96.8(12.0)/ – (–) 25.6(5.2)/ – (–) 25.3(5.8)/ – (–) 23.1(6.1)/–(–) 22.8(6.0)/– (–)	−19.3(1.4) −14.4(1.4) −6.7(0.5) −5.6(0.5) −2.1(0.5) −1.4(0.5)	s	ns	ns
Bošković et al., 2016	Schizophrenia outpatients added to stable dose of haloperidol	≥18	16 weeks	Haloperidol	EPA 0.396g + DHA 0.264g EPA 0.396g + DHA 0.264g Placebo (lactose gelatin) Placebo (lactose gelatin)	18mg 1200IU^c^ 18mg 1200IU^c^	9 9 11 5	Total PANSS	61.7(21.8)/–(–) 51.5(18.4)/– (–) 57.4(15.4)/– (–) 47.0(13.1)/– (–)	– (–) – (–) – (–) – (–)	ns	ns	ns
McGorry et al., 2017	UHR unspecified patients on monotherapy	13–40	26 weeks	CBCM	EPA 0.84g + DHA 0.56g Placebo (paraffin oil)	4.5mg N/A	153 151	Total BPRS Positive BPRS Negative BPRS	73.9(17.7)/– (–) 72.7(14.8)/(–) – (–)/– (–) – (–)/– (–) – (–)/– (–) – (–)/– (–)	−10.3(14.2) −10.4(14.2) −3.3(4.2) −3.4(5.2) −1.98(3.2) −2.2(3.9)	ns	ns	ns
Behdani et al., 2018	Schizophrenia inpatients added to stable dose of clozapine and valproate	18–60	8 weeks	Clozapine and Valproate	EPA 0.72g + DHA 0.48g Placebo (olive oil)	N/A N/A	28 28	Total PANSS	– (–)/– (–) – (–)/– (–)	– (–) – (–)	–	–	–
Nelson et al., 2018^b^	UHR unspecified patients on monotherapy	13–40	3.4 years^f^	CBCM	EPA 0.84g + DHA 0.56g Placebo (paraffin oil)		71 70	Total BPRS	41.6(–)/32.3(–) 41.4(–)/32.1(–)	−9.4(10.1) −9.3(10.0)	ns	–	–
Qiao et al., 2018	Schizophrenia inpatients with symptoms of aggression added to flexible dose of clozapine	18–60	12 weeks	Unspecified antipsychotics	EPA 0.54g + DHA 0.36g Placebo (identical capsule)	N/A 10mg	28 22	Total PANSS Positive PANSS Negative PANSS	87.8(19.3)/61.7(18.5) 89.8(14.4)/63.0(17.0) 25.9(6.0)/– (–) 27.0(4.5)/– (–) 19.4(8.2)/– (–) 19.3(7.7)/– (–)	−26.1(12.6) −23.5(10.3) –(–) –(–) –(–) –(–)	ns	–	–
Robinson et al., 2019	Recent-onset schizophrenia, schizoaffective, schizophreniform, or bipolar inpatients started concurrently with risperidone	15–40	16 weeks	Risperidone	EPA 0.74g + DHA 0.4g Placebo (soybean/corn oil)	4mg N/A	25 25	Total BPRS Positive BPRS Negative BPRS	69.8(11.2)/– (–) 71.3(11.9)/– (–) – (–)/– (–) – (–)/– (–) – (–)/– (–) – (–)/– (–)	−30.7(8.9) −23.5(10.3) −9.5(4.0) −8.0(5.3) 2.5(0.9) −0.80(0.71)	s	ns	ns
Qiao et al., 2020	Schizophrenia inpatients with symptoms of aggression added to stable dose of antipsychotics	34^g^	8 weeks	Unspecified antipsychotics	EPA 0.54g + DHA 0.36g Placebo (identical capsule)	N/A 10mg	32 35	Total PANSS Positive PANSS Negative PANSS	91.5(16.8)/67.4(16.5) 88.4(13.8)/59.9(18.7) 26.5(6.4)/17.2(5.5) 27.09(5.01)/16.0(6.05) 20.8(7.6)/16.6(6.7) 17.9(6.9)/14.7(7.6)	−24.1(16.7) −28.5(16.8) −9.3(6.0) −11.09(5.7) −4.2(7.2) −3.2(7.2)	sdc	sdc	sdc
Tang et al., 2020	Schizophrenia inpatients with metabolic syndrome on long-term olanzapine monotherapy	18–45	12 weeks	Monotherapy	EPA 0.36g + DHA 0.24g Placebo (corn oil)	N/A 100mg	37 35	Total PANSS Positive PANSS Negative PANSS	44.9(7.8)/44.0(6.7) 42.4(9.9)/41.9(6.7) 10.3(3.4)/10.4(3.2) 9.2(2.7)/10.2(2.0) 12.03(3.6)/11.7(3.3) 11.8(4.0)/11.3(3.5)	−0.9(7.3) −0.5(8.8) 0.1(3.3) 1.0(2.4) −0.33(3.5) −0.5(3.8)	ns	ns	ns
Qurashi et al., 2024	At-risk mental state help-seeking outpatients added to stable dose of minocycline	16–35	26 weeks	Minocycline	EPA 0.72g + DHA 0.48g^h^ Placebo (identical capsule)	N/A N/A	65 73	Global CAARMS	14.1(1.7)/8.5(–) 13.8(1.8)/9.0(–)	−5.6(3.8) −4.8(4.1)	–	s	–
Winter-van Rossum et al., 2024	UHR help-seeking outpatients with antipsychotics discontinued within 2 months	13–20	26 weeks	Monotherapy	EPA 0.72g + DHA 0.48g Placebo (identical capsule)	7.6mg N/A	62 65	Total PANSS Positive PANSS Negative PANSS	59.0(13.8)/51.8(19.0) 60.2(15.1)/46.5(14.2) 13.0(3.3)/11.0(4.2) 12.8(3.1)/9.9(3.1) 14.0(5.2)/13.2(6.9) 14.7(5.9)/11.9(6.0)	−7.2(17.0) −13.7(14.7) −2.0(3.8) −2.9(3.1) −0.8(6.2) −2.8(6.0)	ns	ns	ns

Abbreviations: BPRS, brief psychiatric rating scale; CAARMS, comprehensive assessment of at-risk mental state; CBCM, cognitive behavioural case management; DHA, docosahexaenoic acid; EPA, eicosapentaenoic acid; N/A indicates data not applicable; PANSS, positive and negative syndrome scale. - indicates data unavailable; SD, standard deviation.

a Efficacy of omega-3 treatment on symptom improvement for each individual study. T = PANSS total scores; P = PANSS positive subscale; N = PANSS negative subscale; s = significant improvement of symptoms; sdc = significant decline of symptoms; ns = no significant change of symptoms.

b Medium-term/long-term follow-up outcome study from a previous randomized placebo-controlled trial.

c Vitamin E was treated as an active comparison in the study.

d Two different studies were published in a single article.

e Patients received daily dosages of 2 g EPA, 1 g DHA, and 0.3 g alpha-lipoic acid.

f Mean time to follow-up with a range of 1.5–5.7 years.

g Mean age of participants.

h Intervention groups included minocycline, omega-3, combined minocycline, and omega-3 or double placebo. Data presented are for omega-3 and double placebo only.

### Statistical analysis

Mean change in psychometric data was calculated by subtracting post-intervention scores with baseline scores while SD change from baseline was calculated using the following equation: 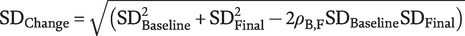 . Correlation coefficient was estimated at 0.5 in the case of an unknown value. Results of mean difference were presented as negative values representing a reduction in psychotic symptoms.

The Cochrane Review Manager version 5.4 was used to evaluate any treatment effect between the omega-3 and control groups. The effect sizes and covariate effects were combined across studies using random-effects meta-analysis models with inverse variance weighting used to summarize the effects across studies and estimate the SMDs and their corresponding 95% confidence intervals (CIs) for continuous outcomes. Finally, the heterogeneity of studies was assessed using the *I* ^2^ statistic before evaluating any publication bias using a funnel plot asymmetry.

## Results

### Literature search

**Figure 1.**
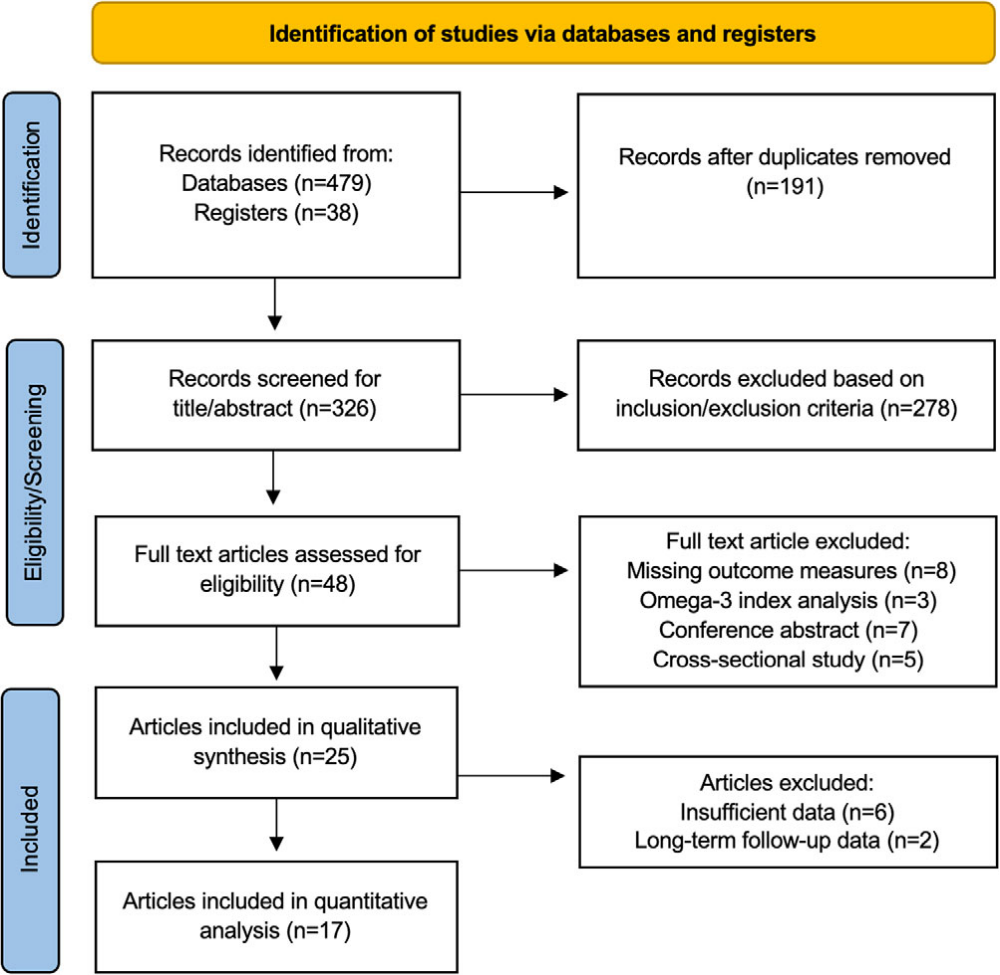
PRISMA 2020 flow diagram. Preferred Reporting Items for Systematic Reviews and Meta-analyses (PRISMA) flowchart. From: Page MJ, McKenzie JE, Bossuyt PM, Boutron I, Hoffmann TC, Mulrow CD, et al. The PRISMA 2020 statement: an updated guideline for reporting systematic reviews. *BMJ.* 2021;372:n71. Doi: 10.1136/bmj.n71. For more information, visit: https://www.prisma-statement.org.

A copy of the PRISMA flow diagram, outlining the search strategy of the literature is presented in Figure 1. The literature search yielded a total of 517 potentially relevant articles. Titles and abstracts were reviewed, and irrelevant articles were discarded. The use of automation filter tools when available were used to refine the literature search to RCTs only. Consequently, 48 full-text articles were examined, with 25 RCTs selected after meeting the inclusion and exclusion criteria. Eight studies were subsequently excluded from the meta-analysis due to insufficient baseline and/or follow-up psychometric scores [19–21, 35–37], and for being duplicate samples of medium or long-term follow-up RCTs [38, 39].

### Selected studies

Included studies evaluated UHR (*n* = 6) [12, 14, 38–41], FEP (*n* = 3) [17, 19, 42], and chronic schizophrenia (*n* = 16) [15, 20–23, 35–37, 43–48, 50] (Table 1). Of the 16 studies examining individuals with schizophrenia, five included individuals either with schizoaffective or schizophreniform disorder [21,23,42,43]. In addition, two studies included participants with bipolar disorder [35, 47] and were deemed eligible for inclusion considering the small number of participants from this cohort [35, 47]. Omega-3 supplementation was administered as an adjunct to stable or flexible doses of antipsychotics with the exception of 6 RCTs using monotherapy only [12, 14, 38, 39, 42, 50]. Clozapine was used as an adjunctive treatment in 6 RCTs [20, 23, 43–45, 48]. However, only a single study provided separate outcome data for this group [20], preventing subgroup analyses of differential treatment response between clozapine and first or second-generation antipsychotics.

The 17 identified studies for the meta-analysis included 1440 participants between 13 and 70 years of age. All studies used either mixed EPA and DHA formulations or EPA-predominant formulations, with only one study including a stratum with a DHA formulation [15]. Consequently, no analysis comparing EPA versus DHA predominant formulations was undertaken.

### Therapeutic efficacy

**Figure 2.**
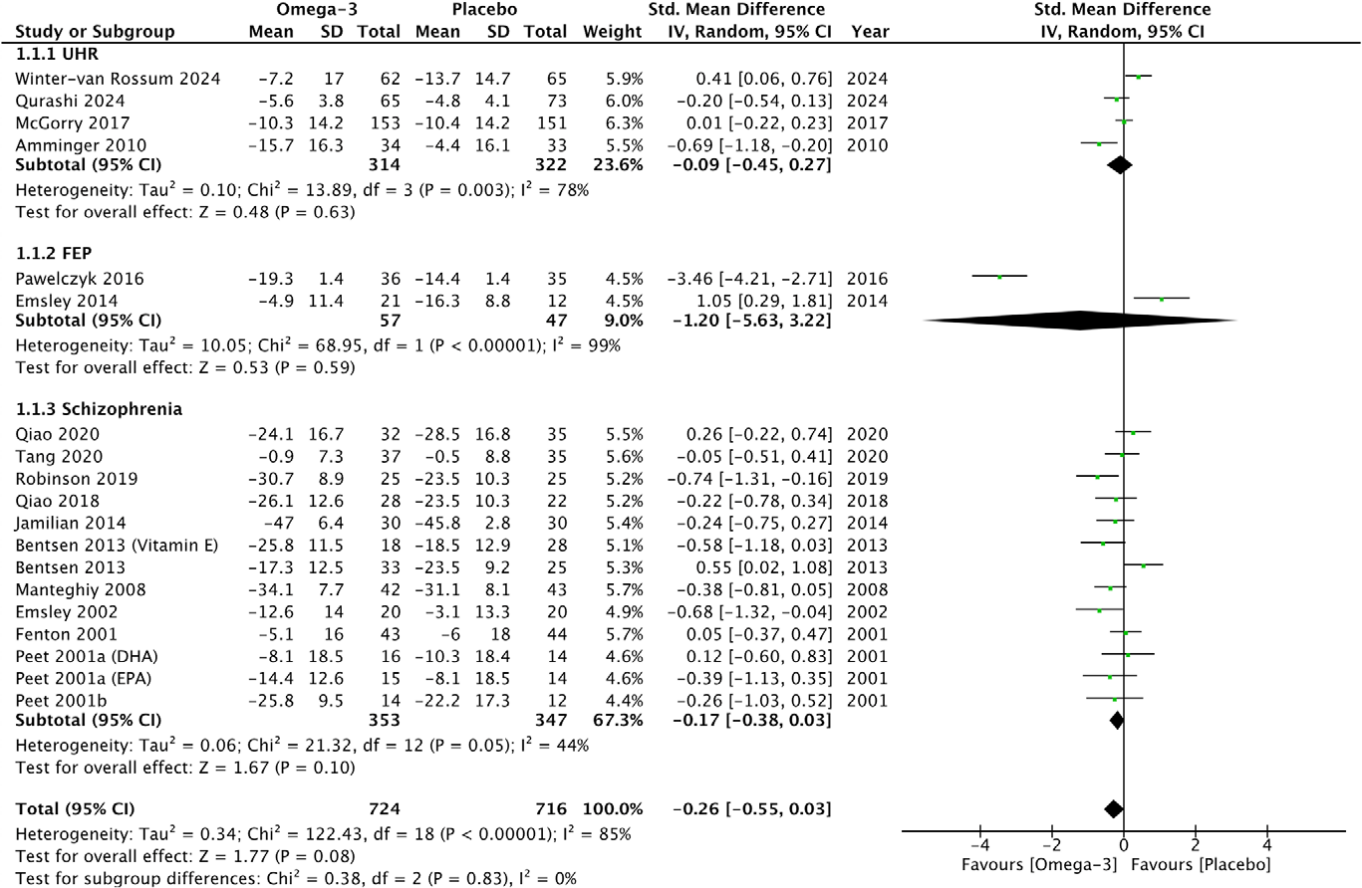
Standardized mean difference for changes in psychopathology from scores on selected psychometric total scale. UHR: ultra-high risk for psychosis; FEP: first-episode psychosis; CI: confidence interval; SD: standard deviation.

**Figure 3.**
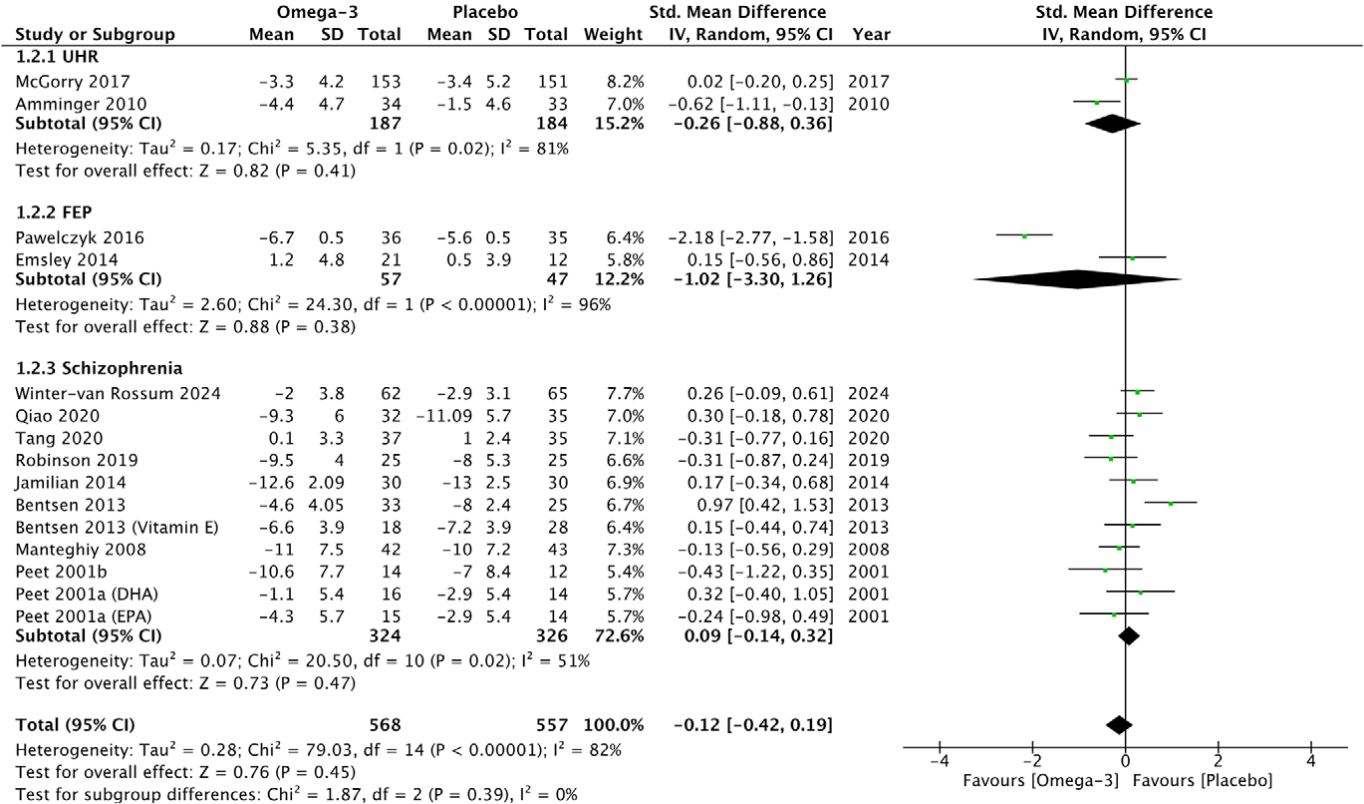
Standardized mean difference for changes in psychopathology from scores on selected psychometric positive subscale. UHR: ultra-high risk for psychosis; FEP: first-episode psychosis; CI: confidence interval; SD: standard deviation.

**Figure 4.**
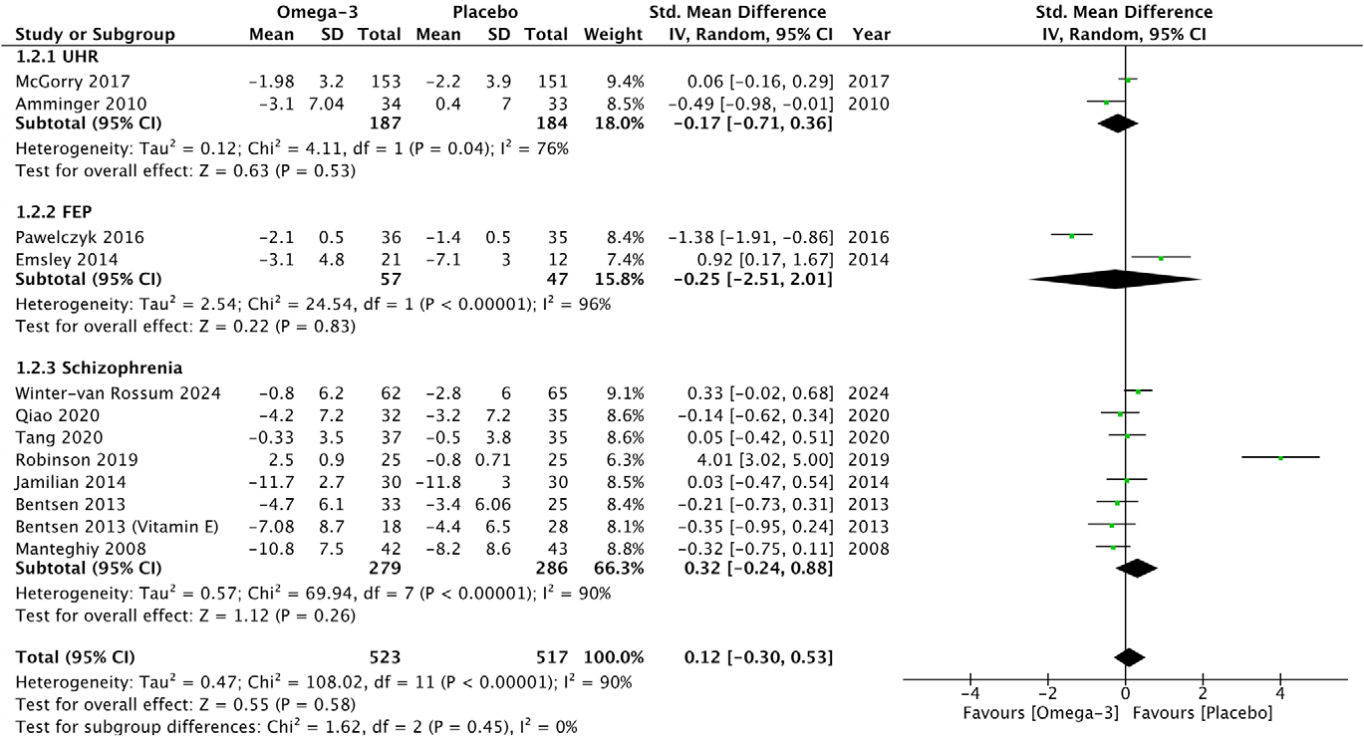
Standardized mean difference for changes in psychopathology from scores on selected psychometric negative subscale. UHR: ultra-high risk for psychosis; FEP: first-episode psychosis; CI: confidence interval; SD: standard deviation.

**Figure 5.**
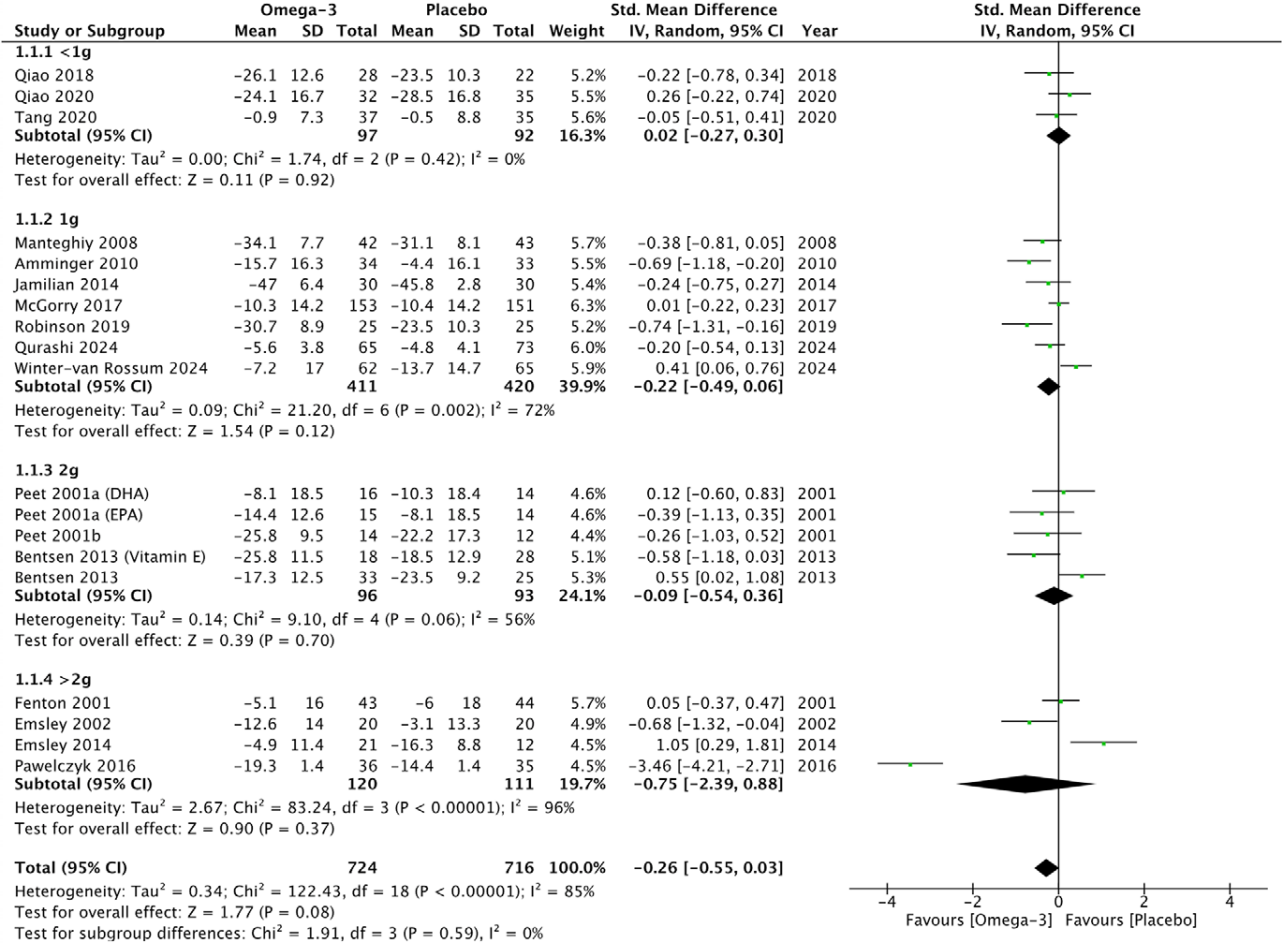
Standardized mean difference for changes in psychopathology from scores on selected psychometric total scale by dosage of omega-3 supplementation. UHR: ultra-high risk for psychosis; FEP: first-episode psychosis; CI: confidence interval; SD: standard deviation.

Omega-3 PUFAs did not demonstrate any significant reduction in psychotic symptoms compared to placebo on psychometric total scales (*G* = −0.26, 95% CI −0.55 to 0.03, *p* = 0.08) (Figure 2), positive subscales (*G* = −0.12, 95% CI −0.42 to 0.19, *p* = 0.45) (Figure 3), and negative subscales (*G* = 0.12 95% CI −0.30 to 0.53, *p* = 0.58) (Figure 4). Subgroup analyses did not demonstrate significant omega-3 treatment efficacy for total symptom scores in UHR (*G* = −0.09, 95% CI −0.45 to 0.27, *p* = 0.63), FEP (*G* = −1.20, 95% CI −5.63 to 3.22, *p* = 0.59), and schizophrenia (*G* = −0.17, 95% CI −0.38 to 0.03, *p* = 0.10) (Figure 2). Similarly, no improvement was found for positive symptom scores across all groups (UHR: *G* = −0.26, 95% CI −0.88 to 0.36, *p* = 0.41), (FEP: *G* = −1.02, 95% CI −3.30 to 1.26, *p* = 0.38) and (schizophrenia: *G* = 0.09, 95% CI −0.14 to 0.32, *p* = 0.47) (Figure 3) nor did for negative symptoms scores (UHR: *G* = −0.17, 95% CI −0.71 to 0.36, *p* = 0.53), (FEP: *G* = −0.25, 95% CI −2.51 to 2.01, *p* = 0.83) and (schizophrenia: *G* = 0.32, 95% CI −0.30 to 0.53, *p* = 0.26) (Figure 4). Subgroup analyses of omega-3 PUFA dosages did not demonstrate any statistical overall effect between improvement in psychopathology and dosages administered across all stages of psychosis (Figure 5).

### Quality assessment

**Figure 6.**
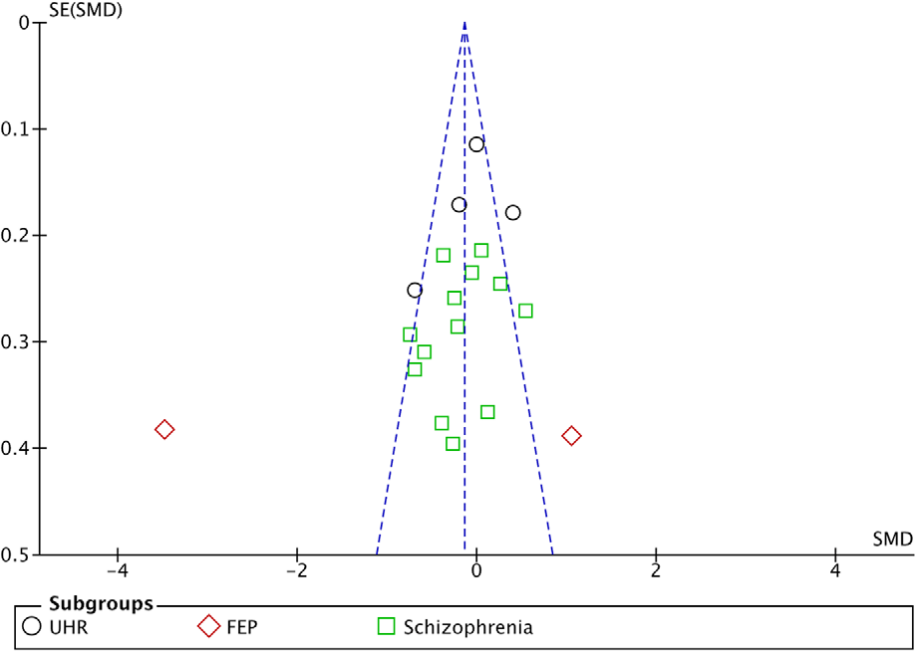
Funnel plot of standard error (SE) by SMD.

The quality assessment for all the RCTs is presented in Table 2. The overall risk of bias in the individual studies was low as assessed by the Cochrane Risk of Bias Assessment Tool with the exception of 4 studies [22, 36, 45, 46]. Some concerns in the randomization process, specifically regarding knowledge of the forthcoming interventions allocation by investigators and/or participants, were revealed for nine studies [14,22,36,43–47] and for missing outcome measures for two studies [36, 43]. High risk of bias due to missing outcome data was also noted for four studies [22, 36, 45, 46]. Publication bias for the meta-analysis RCTs was assessed qualitatively by funnel plot asymmetry (Figure 6). It was estimated that a number of studies with negative effect sizes might never have been published; however, this plot evaluated all studies, with the three studies demonstrating a more positive therapeutic effect noted to include individuals at UHR for psychosis or with a FEP.

**Table 2.** ROB2 risk of bias assessment for individual randomized placebo-controlled trials.

Study reference	Domain 1	Domain 2	Domain 3	Domain 4	Domain 5
Fenton et al., 2001	Some concerns	Low risk	Low risk	Some concerns	Low risk
Peet et al., 2001a	Low risk	Low risk	Low risk	Low risk	Low risk
Peet et al., 2001b	Low risk	Low risk	Low risk	Low risk	Low risk
Peet & Horrobin., 2002	Low risk	Low risk	Low risk	Low risk	Low risk
Emsley et al., 2002	Some concerns	Low risk	Low risk	Low risk	Low risk
Emsley et al., 2006	Low risk	Low risk	Low risk	Low risk	Low risk
Berger et al., 2007	Low risk	Low risk	Low risk	Low risk	Some concerns
Manteghiy et al., 2008	Some concerns	Low risk	High risk	Low risk	Low risk
Amminger et al., 2010	Low risk	Low risk	Low risk	Low risk	Low risk
Faghihi et al., 2012	Low risk	Low risk	Low risk	Low risk	Low risk
Bentsen et al., 2013	Low risk	Low risk	Some concerns	Low risk	Some concerns
Emsley et al., 2014	Low risk	Low risk	Low risk	Low risk	Low risk
Jamilian et al., 2014	Some concerns	Low risk	High risk	Low risk	Low risk
Amminger et al., 2015	Low risk	Low risk	Low risk	Low risk	Low risk
Pawełczyk et al., 2016	Low risk	Low risk	Low risk	Low risk	Low risk
Bošković et al., 2016	Some concerns	Low risk	High risk	Some concerns	Low risk
McGorry et al., 2017	Some concerns	Low risk	Low risk	Low risk	Low risk
Behdani et al., 2018	Low risk	Low risk	Low risk	Low risk	Low risk
Nelson et al., 2018	Low risk	Low risk	Low risk	Low risk	Low risk
Qiao et al., 2018	Some concerns	Low risk	High risk	Low risk	Low risk
Robinson et al., 2019	Some concerns	Low risk	Low risk	Low risk	Low risk
Qiao et al., 2020	Low risk	Low risk	Low risk	Low risk	Low risk
Tang et al., 2020	Low risk	Low risk	Low risk	Low risk	Low risk
Qurashi et al., 2024	Low risk	Low risk	Some concerns	Low risk	Low risk
Winter-van Rossum et al., 2024	Low risk	Low risk	Low risk	Low risk	Low risk

*Note*: Domain 1: Risk of bias arising from the randomization process; Domain 2: Risk of bias due to deviations from the intended interventions; Domain 3: Risk of bias due to missing outcome data; Domain 4: Risk of bias in measuring of the outcome; Domain 5: Risk of bias in selection of the reported results.

## Discussion

The meta-analysis evaluated the efficacy of omega-3 supplementation in UHR population and schizophrenia patients from all previously published RCTs. Omega-3 PUFAs supplementation did not reveal clinical efficacy for psychosis when compared to placebo. A trend towards improvement in total psychotic symptoms was however noted (*p* = 0.08). Subgroup analyses did not demonstrate any beneficial effect on both positive and negative symptoms across all groups.

In line with previous research, our findings demonstrate that omega-3 PUFAs supplementation is not recommended as a treatment for acute exacerbation of chronic schizophrenia nor for prevention of relapse [25, 36, 43, 48]. Additionally, our findings are in line with recent reports that found no beneficial effect of omega-3 supplements in UHR population [14, 40, 41] although in contradiction with the results from Amminger and colleagues [12]. In light with the accumulating evidence of negative findings, these past positive findings are complex to understand. It is suggested that factors such as improvement in nonpharmacological treatments, comedication, illness severity, and a recent decline in transition rates may contribute to this discrepancy [41, 50]. The most effective dosage of omega-3 PUFAs was 1 g when compared to the other dosages. No optimal omega-3 PUFAs dosage could however be established as no superior statistical effect was found between administration of <1 g, 1g, 2 g, >2 g across all groups. The EPA/DHA dosage and content needed for treatment efficacy has yet to be confirmed and dose-ranging studies are currently limited.

Our findings should be considered in light of some limitations. First, the sample sizes and patient populations in the included RCTs were not consistent, particularly for RCTs of FEP which may have affected the validity and generalizability of the outcomes. Furthermore, baseline characteristics varied across all studies when controlling for diagnostic tools, severity of illness and omega-3 formulations. Publication bias was also found for a number of studies and included non-reporting of outcome measures and/or psychopathology domains. In addition, the effect of adjunctive antipsychotics or nonpharmacological treatments such as CBCM could not be excluded. Because no specification of adjunctive antipsychotics was provided on a number of RCTs, this prevented further analyses for such effect. The outcome measures in this study had relatively small to medium effect sizes, suggesting limited practical significance of the results. Finally, significant heterogeneity in findings was noted. This is suggested, however, to be related to heterogeneous sample populations and variable omega-3 PUFA formulations utilized.

## Conclusion

The current evidence supports initially reported results on the use of omega-3 PUFAs in the treatment of symptom severity in prodromal/chronic schizophrenia patients and more recently in populations at high-risk states for psychosis. Omega-3 supplementation is not considered to be a suitable early treatment strategy for psychotic disorders and future studies in this line of research are not suggested.

## Supporting information

Rossier and Hallahan supplementary material Rossier and Hallahan supplementary material

## Data Availability

All data supporting the findings of this study are available within the article.

## Supplementary material

The supplementary material for this article can be found at http://doi.org/10.1192/j.eurpsy.2024.1804.

## References

[1] Carrion RE, McLaughlin D, Goldberg TE, Auther AM, Olsen RH, Olvet DM, et al. Prediction of functional outcome in individuals at clinical high risk for psychosis. JAMA Psychiatry. 2013;70 (11):1133–42. 24006090 10.1001/jamapsychiatry.2013.1909 PMC4469070

[2] Iasevoli F, Giordano S, Balletta R, Latte G, Formato MV, Prinzivalli E, et al. Treatment resistant schizophrenia is associated with the worst community functioning among severely-ill highly-disabling psychiatric conditions and is the most relevant predictor of poorer achievements in functional milestones. Prog Neuro-Psychopharmacol Biol Psychiatry. 2016;65:34–48. 10.1016/j.pnpbp.2015.08.010 26320028

[3] Horrobin DF, Glen AI, Vaddadi K. The membrane hypothesis of schizophrenia. Schizophr Res. 1994;13 (3):195–207. 7841132 10.1016/0920-9964(94)90043-4

[4] Horrobin DF. The membrane phospholipid hypothesis as a biochemical basis for the neurodevelopmental concept of schizophrenia. Schizophr Res. 1998;30 (3):193–208. 9589514 10.1016/s0920-9964(97)00151-5

[5] Nuss P, Tessier C, Ferreri F, De Hert M, Peuskens J, Trugnan G, et al. Abnormal transbilayer distribution of phospholipids in red blood cell membranes in schizophrenia. Psychiatry Res. 2009;169 (2):91–6. 19646766 10.1016/j.psychres.2009.01.009

[6] Yao JK, Leonard S, Reddy RD. Membrane phospholipid abnormalities in postmortem brains from schizophrenic patients. Schizophr Res. 2000;42 (1):7–17. 10706981 10.1016/s0920-9964(99)00095-x

[7] Rice SM, Schafer MR, Klier C, Mossaheb N, Vijayakumar N, Amminger GP. Erythrocyte polyunsaturated fatty acid levels in young people at ultra-high risk for psychotic disorder and healthy adolescent controls. Psychiatry Res. 2015;228 (1):174–6. 25979466 10.1016/j.psychres.2015.04.036

[8] Amminger GP, Schafer MR, Klier CM, Slavik JM, Holzer I, Holub M, et al. Decreased nervonic acid levels in erythrocyte membranes predict psychosis in help-seeking ultra-high-risk individuals. Mol Psychiatry. 2012;17 (12):1150–2. 22182937 10.1038/mp.2011.167

[9] Reddy RD, Keshavan MS, Yao JK. Reduced red blood cell membrane essential polyunsaturated fatty acids in first episode schizophrenia at neuroleptic-naive baseline. Schizophr Bull. 2004;30 (4):901–11. 15957200 10.1093/oxfordjournals.schbul.a007140

[10] Parletta N, Zarnowiecki D, Cho J, Wilson A, Procter N, Gordon A, et al. People with schizophrenia and depression have a low omega-3 index. Prostaglandins Leukot Essent Fatty Acids. 2016;110:42–7. 27255642 10.1016/j.plefa.2016.05.007

[11] McNamara RK, Jandacek R, Rider T, Tso P, Hahn CG, Richtand NM, et al. Abnormalities in the fatty acid composition of the postmortem orbitofrontal cortex of schizophrenic patients: Gender differences and partial normalization with antipsychotic medications. Schizophr Res. 2007;91 (1-3):37–50. 17236749 10.1016/j.schres.2006.11.027 PMC1853256

[12] Amminger GP, Schafer MR, Papageorgiou K, Klier CM, Cotton SM, Harrigan SM, et al. Long-chain omega-3 fatty acids for indicated prevention of psychotic disorders: A randomized, placebo-controlled trial. Arch Gen Psychiatry. 2010;67 (2):146–54. 20124114 10.1001/archgenpsychiatry.2009.192

[13] Mossaheb N, Schafer MR, Schlogelhofer M, Klier CM, Smesny S, McGorry PD, et al. Predictors of longer-term outcome in the Vienna omega-3 high-risk study. Schizophr Res. 2018;193:168–72. 28823721 10.1016/j.schres.2017.08.010

[14] McGorry PD, Nelson B, Markulev C, Yuen HP, Schafer MR, Mossaheb N, et al. Effect of omega-3 polyunsaturated fatty acids in young people at ultrahigh risk for psychotic disorders: The NEURAPRO randomized clinical trial. JAMA Psychiatry. 2017;74 (1):19–27. 27893018 10.1001/jamapsychiatry.2016.2902

[15] Peet M, Brind J, Ramchand CN, Shah S, Vankar GK. Two double-blind placebo-controlled pilot studies of eicosapentaenoic acid in the treatment of schizophrenia. Schizophr Res. 2001;49 (3):243–51. 11356585 10.1016/s0920-9964(00)00083-9

[16] Berger GE, Wood SJ, Wellard RM, Proffitt TM, McConchie M, Amminger GP, et al. Ethyl-eicosapentaenoic acid in first-episode psychosis. A 1H-MRS study. Neuropsychopharmacology. 2008;33 (10):2467–73. 18199999 10.1038/sj.npp.1301628

[17] Pawelczyk T, Grancow-Grabka M, Kotlicka-Antczak M, Trafalska E, Pawelczyk A. A randomized controlled study of the efficacy of six-month supplementation with concentrated fish oil rich in omega-3 polyunsaturated fatty acids in first episode schizophrenia. J Psychiatr Res. 2016;73:34–44. 26679763 10.1016/j.jpsychires.2015.11.013

[18] Pawelczyk T, Grancow-Grabka M, Zurner N, Pawelczyk A. Omega-3 fatty acids reduce cardiometabolic risk in first-episode schizophrenia patients treated with antipsychotics: Findings from the OFFER randomized controlled study. Schizophr Res. 2021;230:61–8. 33684737 10.1016/j.schres.2021.02.012

[19] Berger GE, Proffitt TM, McConchie M, Yuen H, Wood SJ, Amminger GP, et al. Ethyl-eicosapentaenoic acid in first-episode psychosis: A randomized, placebo-controlled trial. J Clin Psychiatry. 2007;68 (12):1867–75. 18162017 10.4088/jcp.v68n1206

[20] Peet M, Horrobin DF, Group EEMS. A dose-ranging exploratory study of the effects of ethyl-eicosapentaenoate in patients with persistent schizophrenic symptoms. J Psychiatr Res. 2002;36 (1):7–18. 11755456 10.1016/s0022-3956(01)00048-6

[21] Emsley R, Niehaus DJ, Koen L, Oosthuizen PP, Turner HJ, Carey P, et al. The effects of eicosapentaenoic acid in tardive dyskinesia: A randomized, placebo-controlled trial. Schizophr Res. 2006;84 (1):112–20. 16632329 10.1016/j.schres.2006.03.023

[22] Manteghiy A, Shakeri MT, Koohestani L, Salari E. Beneficial antipsychotic effects of omega-3 fatty acids add-on therapy for the pharmacological management of patients with schizophrenia. Iran J Psychiatry Behav Sci. 2008;2 (2):35–40.

[23] Bentsen H, Osnes K, Refsum H, Solberg DK, Bohmer T. A randomized placebo-controlled trial of an omega-3 fatty acid and vitamins E+C in schizophrenia. Transl Psychiatry. 2013;3 (12):e335. 24346133 10.1038/tp.2013.110 PMC3906471

[24] Goh KK, Chen CY, Chen CH, Lu ML. Effects of omega-3 polyunsaturated fatty acids supplements on psychopathology and metabolic parameters in schizophrenia: A meta-analysis of randomized controlled trials. J Psychopharmacol. 2021;35 (3):221–35. 33586517 10.1177/0269881120981392

[25] Chen AT, Chibnall JT, Nasrallah HA. A meta-analysis of placebo-controlled trials of omega-3 fatty acid augmentation in schizophrenia: Possible stage-specific effects. Ann Clin Psychiatry. 2015;27 (4):289–96. 26554370

[26] Fusar-Poli P, Berger G. Eicosapentaenoic acid interventions in schizophrenia: Meta-analysis of randomized, placebo-controlled studies. J Clin Psychopharmacol. 2012;32 (2):179–85. 22367656 10.1097/JCP.0b013e318248b7bb

[27] Xu X, Shao G, Zhang X, Hu Y, Huang J, Su Y, et al. The efficacy of nutritional supplements for the adjunctive treatment of schizophrenia in adults: A systematic review and network meta-analysis. Psychiatry Res. 2022;311:114500. 35287043 10.1016/j.psychres.2022.114500

[28] Devoe DJ, Farris MS, Townes P, Addington J. Attenuated psychotic symptom interventions in youth at risk of psychosis: A systematic review and meta-analysis. Early Interv Psychiatry. 2019;13 (1):3–17. 29749710 10.1111/eip.12677 PMC6230498

[29] Devoe DJ, Peterson A, Addington J. Negative symptom interventions in youth at risk of psychosis: A systematic review and network meta-analysis. Schizophr Bull. 2018;44 (4):807–23. 29069511 10.1093/schbul/sbx139 PMC6007754

[30] Page MJ, McKenzie JE, Bossuyt PM, Boutron I, Hoffmann TC, Mulrow CD, et al. The PRISMA 2020 statement: An updated guideline for reporting systematic reviews. BMJ. 2021;372:n71. 33782057 10.1136/bmj.n71 PMC8005924

[31] American Psychiatric Association. Diagnostic and statistical manual of mental disorders. 5th ed. American Psychiatric Press; 2013.

[32] World Health Organization. The ICD-10 classification of mental and behavioural disorders: Clinical descriptions and diagnostic guidelines. 2nd ed. WHO; 2004.

[33] Yung AR, Yuen HP, McGorry PD, Phillips LJ, Kelly D, Dell’Olio M, et al. Mapping the onset of psychosis: The comprehensive assessment of at-risk mental states. Aust N Z J Psychiatry. 2005;39 (11–12):964–71. 16343296 10.1080/j.1440-1614.2005.01714.x

[34] Miller TJ, McGlashan TH, Rosen JL, Cadenhead K, Cannon T, Ventura J, et al. Prodromal assessment with the structured interview for prodromal syndromes and the scale of prodromal symptoms: Predictive validity, interrater reliability, and training to reliability. Schizophr Bull. 2003;29 (4):703–15. 14989408 10.1093/oxfordjournals.schbul.a007040

[35] Faghihi T, Jahed A, Mahmoudi-Gharaei J, Sharifi V, Akhondzadeh S, Ghaeli P. Role of Omega-3 fatty acids in preventing metabolic disturbances in patients on olanzapine plus either sodium valproate or lithium: A randomized double-blind placebo-controlled trial. Daru. 2012;20 (1):43. 23351198 10.1186/2008-2231-20-43 PMC3555734

[36] Boskovic M, Vovk T, Koprivsek J, Plesnicar BK, Grabnar I. Vitamin E and essential polyunsaturated fatty acids supplementation in schizophrenia patients treated with haloperidol. Nutr Neurosci. 2016;19 (4):156–61. 25056532 10.1179/1476830514Y.0000000139

[37] Behdani F, Roudbaraki SN, Saberi-Karimian M, Tayefi M, Hebrani P, Akhavanrezayat A, et al. Assessment of the efficacy of omega-3 fatty acids on metabolic and inflammatory parameters in patients with schizophrenia taking clozapine and sodium valproate. Psychiatry Res. 2018;261:243–7. 29329042 10.1016/j.psychres.2017.12.028

[38] Nelson B, Amminger GP, Yuen HP, Markulev C, Lavoie S, Schafer MR, et al. NEURAPRO: A multi-Centre RCT of omega-3 polyunsaturated fatty acids versus placebo in young people at ultra-high risk of psychotic disorders-medium-term follow-up and clinical course. NPJ Schizophr. 2018;4 (1):11. 29941938 10.1038/s41537-018-0052-x PMC6018097

[39] Amminger GP, Schafer MR, Schlogelhofer M, Klier CM, McGorry PD. Longer-term outcome in the prevention of psychotic disorders by the Vienna omega-3 study. Nat Commun. 2015;6:7934. 26263244 10.1038/ncomms8934 PMC4918317

[40] Qurashi I, Chaudhry IB, Khoso AB, Omair Husain M, Hafeez D, Kiran T, et al. A randomised double-blind placebo-controlled trial of minocycline and/or omega-3 fatty acids added to treatment as usual for at risk mental states: The NAYAB study. Brain Behav Immun. 2024;115:609–16. 37924960 10.1016/j.bbi.2023.10.025

[41] Winter-van Rossum I, Slot MIE, van Hell HH, Bossong MG, Berger G, Aschauer H, et al. Effectiveness of omega-3 fatty acids versus placebo in subjects at ultra-high risk for psychosis: The purpose randomized clinical trial. Schizophr Bull. 2024. 10.1093/schbul/sbae186 PMC12236319 39450759

[42] Emsley R, Chiliza B, Asmal L, du Plessis S, Phahladira L, van Niekerk E, et al. A randomized, controlled trial of omega-3 fatty acids plus an antioxidant for relapse prevention after antipsychotic discontinuation in first-episode schizophrenia. Schizophr Res. 2014;158 (1–3):230–5. 24996507 10.1016/j.schres.2014.06.004

[43] Fenton WS, Dickerson F, Boronow J, Hibbeln JR, Knable M. A placebo-controlled trial of omega-3 fatty acid (ethyl eicosapentaenoic acid) supplementation for residual symptoms and cognitive impairment in schizophrenia. Am J Psychiatry. 2001;158 (12):2071–4. 11729030 10.1176/appi.ajp.158.12.2071

[44] Emsley R, Myburgh C, Oosthuizen P, van Rensburg SJ. Randomized, placebo-controlled study of ethyl-eicosapentaenoic acid as supplemental treatment in schizophrenia. Am J Psychiatry. 2002;159 (9):1596–8. 12202284 10.1176/appi.ajp.159.9.1596

[45] Jamilian H, Solhi H, Jamilian M. Randomized, placebo-controlled clinical trial of omega-3 as supplemental treatment in schizophrenia. Glob J Health Sci. 2014;6(7 Spec No):103–8. 25363186 10.5539/gjhs.v6n7p103 PMC4796520

[46] Qiao Y, Mei Y, Han H, Liu F, Yang XM, Shao Y, et al. Effects of omega-3 in the treatment of violent schizophrenia patients. Schizophr Res. 2018;195:283–5. 28830741 10.1016/j.schres.2017.08.026

[47] Robinson DG, Gallego JA, John M, Hanna LA, Zhang JP, Birnbaum ML, et al. A potential role for adjunctive omega-3 polyunsaturated fatty acids for depression and anxiety symptoms in recent onset psychosis: Results from a 16 week randomized placebo-controlled trial for participants concurrently treated with risperidone. Schizophr Res. 2019;204:295–303. 30241990 10.1016/j.schres.2018.09.006 PMC6402999

[48] Qiao Y, Liu CP, Han HQ, Liu FJ, Shao Y, Xie B. No impact of omega-3 fatty acid supplementation on symptoms or hostility among patients with schizophrenia. Front Psych. 2020;11:312. 10.3389/fpsyt.2020.00312 PMC7186329 32372988

[49] Tang W, Wang Y, Xu F, Fan W, Zhang Y, Fan K, et al. Omega-3 fatty acids ameliorate cognitive dysfunction in schizophrenia patients with metabolic syndrome. Brain Behav Immun. 2020;88:529–34. 32304881 10.1016/j.bbi.2020.04.034

[50] Hartmann JA, Yuen HP, McGorry PD, Yung AR, Lin A, Wood SJ, et al. Declining transition rates to psychotic disorder in “ultra-high risk” clients: Investigation of a dilution effect. Schizophr Res. 2016;170 (1):130–6. 26673973 10.1016/j.schres.2015.11.026

